# Electrodeposition of CdTe Thin Films for Solar Energy Water Splitting

**DOI:** 10.3390/ma13071536

**Published:** 2020-03-27

**Authors:** Jun Ling, Xulei Zhang, Ting Mao, Lei Li, Shilin Wang, Meng Cao, Jijun Zhang, Haozhi Shi, Jian Huang, Yue Shen, Linjun Wang

**Affiliations:** 1 State Key Laboratory of Nuclear Power Safety Monitoring Technology and Equipment, China Nuclear Power Engineering Co., Ltd, Shenzhen 518124, China; lingjun_315@sjtu.edu.cn (J.L.); maoting@cgnpc.com.cn (T.M.); lilei3@cgnpc.com.cn (L.L.); 2 Department of Automation, Shanghai Jiao Tong University, Shanghai 200240, China; 3 School of Materials Science and Engineering, Shanghai University, Shanghai 200072, China; 18800207664@163.com (X.Z.); 13818737657@163.com (S.W.); zhangjijun222@shu.edu.cn (J.Z.); shihaozhi@shu.edu.cn (H.S.); jianhuang@shu.edu.cn (J.H.); yueshen@shu.edu.cn (Y.S.); ljwang@shu.edu.cn (L.W.)

**Keywords:** CdTe, photoelectrochemical, electrochemical deposition, PEC properties

## Abstract

CdTe thin films have been prepared by electrochemical deposition. The morphological, structural, and optical properties of CdTe thin films deposited with different deposition time were investigated, and the influence of film thickness on the photoelectric characteristics of CdTe thin films was studied. At the deposition time of 1.5 h, CdTe thin films had good optical properties and the photocurrent reached 20 μAcm^−2^. Furthermore, the Pt/CdS/CdTe/FTO structure was prepared to improve its PEC stability and the photocurrent of 240 μAcm^−2^ had been achieved.

## 1. Introduction

Energy shortage has become the primary problem hindering economic development and world peace and is a focus of attention of all countries in the world. Traditional fossil energy is not only limited, but also has released a great deal of pollution to the environment. As a kind of clean and renewable energy, solar energy has attracted a lot of attention. By utilizing solar energy, the photoelectrochemical (PEC) splitting of water can directly generate hydrogen in a relatively simple process [1]. Cadmium telluride (CdTe) has a number of attractive properties as a photocathode material for PEC water splitting and absorbing materials in photovoltaic cells [2]. It has a direct band gap of 1.45 eV and high light absorption coefficient, which can reach 10^4^ cm^−1^ in the visible light range [3,4,5]. 

CdTe thin films can be prepared with various methods, such as near space sublimation [6], magnetron sputtering [7], vapor transport deposition [8], and so on. Among these methods, electrochemical deposition is considered to be an ideal method for mass production of CdTe films with easy operation and high material utilization [9,10]. There are several advantages for the electrodeposition process. For example, it is easy to operate without high vacuum or a high temperature environment. Both p-type and n-type CdTe have been easily deposited and electrodeposition potential was found to be the key factor [11]. Novel morphologies, such as CdTe nanowires, can be deposited easily by electrochemical deposition method [12,13]. ZnO/CdTe core–shell nanotube arrays have also been synthesized by using a simple two-step electrochemical deposition strategy for solar energy water splitting applications [14]. 

Even though electrodeposited CdTe thin films and their PEC properties have been reported [15], the structure of CdTe photocathode still needs optimization for solar energy water splitting application. In fact, photoelectrochemical properties of CdTe can be enhanced by preparation of a CdTe/CdS PN junction, which is contributive to the separation of photo-generated carriers [2]. A CdS layer can be easily prepared on CdTe thin films. In this work, CdTe thin films were prepared by electrochemical deposition method. The effect of deposition time to the physical and photoelectric properties of CdTe thin films was studied. The PEC response properties of Pt/CdS/CdTe/FTO structure were investigated under illumination of AM 1.5 G, which will be contributive to expand their applications on photoelectric devices.

## 2. Experimental Details

### 2.1. Materials

Sodium tellurite (Na_2_TeO_3_, ≥99.99%), cadmium sulfate (CdSO_4_·8/3H_2_O, ≥99%), cadmium chloride (CdCl_2_·2.5H_2_O, ≥99%), and trisodium citrate (Na_3_C_6_H_5_O_7_·2H_2_O, ≥99%) were purchased from Sinopharm chemical LTD (Shanghai). In addition, sulfuric acid (98%) was used to adjust the pH levels of reaction solution. All solutions were prepared by using distilled water. 

### 2.2. Synthesis

Before depositing the CdTe films, FTO substrates were ultrasonically cleaned by acetone, ethanol, and distilled water for 30 min, respectively. Cleaned FTO substrates were dried with N_2_ and then placed in the oven for drying. Then, 0.1 mmol Na_2_TeO_3_, 1 mmol CdSO_4_·8/3H_2_O, and 3 mmol Na_3_C_6_H_5_O_7_·2H_2_O were first dissolved in 100 mL distilled water under magnetic stirring. Here, Na_3_C_6_H_5_O_7_·2H_2_O was mainly used to control the deposition rate [16]. The pH level of the solution was adjusted to 2 by adding diluted sulfuric acid.

CdTe films were prepared by electrochemical deposition with a three-electrode configuration. In this arrangement, FTO glass, Ag/AgCl in a saturated aqueous KCl solution, and a Pt wire served as the working, reference, and counter electrodes, respectively. They were connected to the electrochemical working station. The magnetic rotor in the solution was set at about 80 r/min, the deposition potential was set to −0.6 V [17], and deposition times were set to 1 h, 1.5 h, 2 h, 2.5 h, respectively. After the deposition, the as-deposited thin films were rinsed by distilled water, dried, and then collected. Saturated CdCl_2_ ethanol solution was dropped onto the surface of CdTe thin films. An additional annealing process was performed to CdTe thin films with CdCl_2_ ethanol solution at 350 °C in a vacuum for 1 hour. 

### 2.3. The Preparation of Pt/CdS/CdTe/FTO Structures

A layer of CdS film was grown on CdTe by chemical bath deposition method [18]. A total of 0.015 M CdSO_4_ and 1.5 M thiourea solutions were first prepared, respectively. Thirty-four milliliters of deionized water, 5 mL CdSO_4_ solution, 5 mL thiourea solution, and 6.5 mL ammonia water were put into a beaker. Then, a CdTe film was deposited on FTO and inserted into the solution maintaining at 60 °C for 8 min. After deposition, the sample was cleaned by distilled water and then dried in the air at room temperature. A layer of 5 nm Pt was sputtered on the CdS surface by using SCD 500 sputter coater (Bal-Tec, Capovani Brothers Inc., Scotia, NY, USA). The sputtering started when the vacuum was lower than 10^−5^ mbar. The sputtering speed was set at 0.3 nm/s and the sputtering time was 17 s.

### 2.4. Characterizations

Structural properties and phase purities of CdTe thin film were examined by X-ray diffraction (XRD, D/MAX2550, Rigaku Inc, Tokyo, Japan, with Cu-Kα radiation, λ = 0.15 nm) and Raman spectra (JY-H800UV, Horiba, Ltd, Kyoto, Japan). Morphologies and compositions of CdTe thin films were determined by scanning electron microscopy (SEM, FEI Sirion 200, Fei Company, Hillsboro, OR, USA). The optical properties were studied by using a UV-vis spectrophotometer (Jasco UV-570, Jasco Inc., Tokyo, Japan). Under illumination of AM 1.5 G (100 mWcm^−2^), the PEC properties of CdTe/FTO and Pt/CdS/CdTe/FTO were studied in a 1 M Na_2_SO_4_ (pH = 1) solution. A CHI660B electrochemical workstation (Chinese Science Day Ltd., Beijing, China) was used. A Pt plate and an Ag/AgCl rod were used as counter-electrode and reference electrode, respectively.

## 3. Results and Discussions

### 3.1. Morphologies and Compositions of Deposited CdTe Thin Films

**Figure 1:**
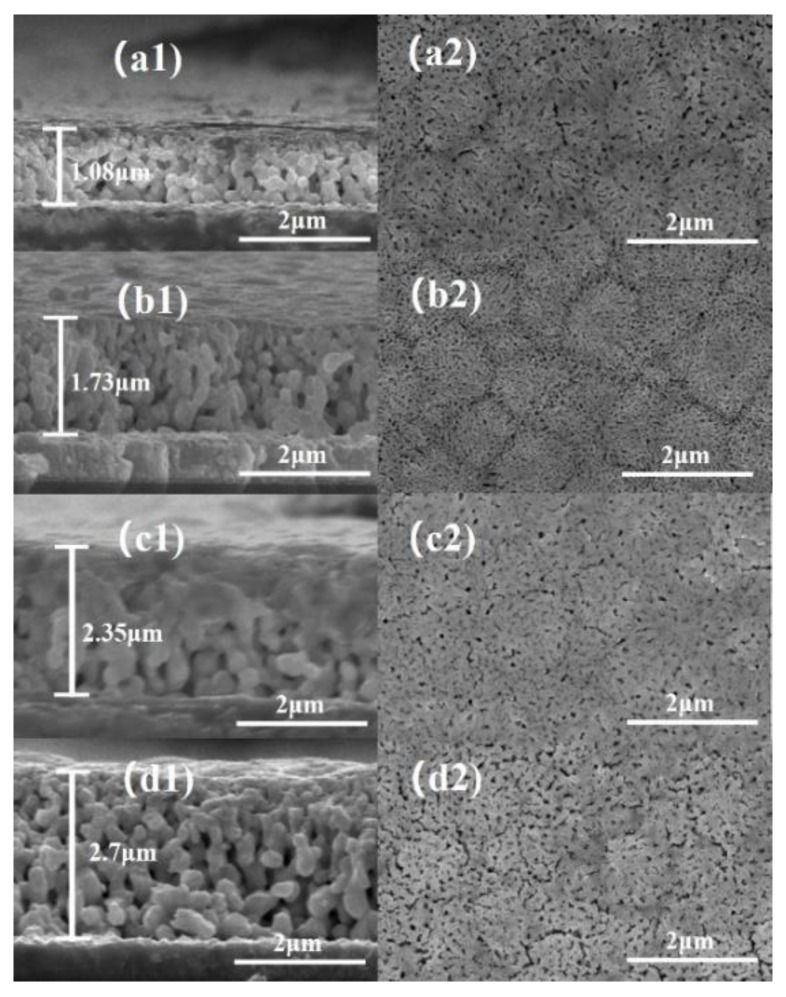
(**a1**–**d1**) Cross-sectional and (**a2**–**d2**) top view SEM images of CdTe thin films grown on FTO substrates with deposition times of 1 h, 1.5 h, 2 h, and 2.5 h, respectively.

The morphological properties of CdTe thin films were tested by SEM. Figure 1a1–d1 proves that the thicknesses of the CdTe thin films are increased with deposition time, going from approximately 1.08 μm to 2.70 μm. The cross-section morphological images indicate that some islanding are formed and seem to be buried by the high deposition rate, and some restricted particles with definite sizes are also formed and combined to form denser layers.

There are some clusters and porous structures at the surface of the deposited CdTe thin films in Figure 1a2–d2. The composition ratios of deposited CdTe thin films were estimated by EDS measurements, as shown in Table 1. 

Even though the CdCl_2_ annealing treatment can make Te in the film combine with Cd from CdCl_2_ to form CdTe, the ratios of Cd/Te of all the samples still indicate that Te-rich CdTe thin films were obtained in our work.

### 3.2. Structural Properties of Deposited CdTe Thin Films 

**Figure 2:**
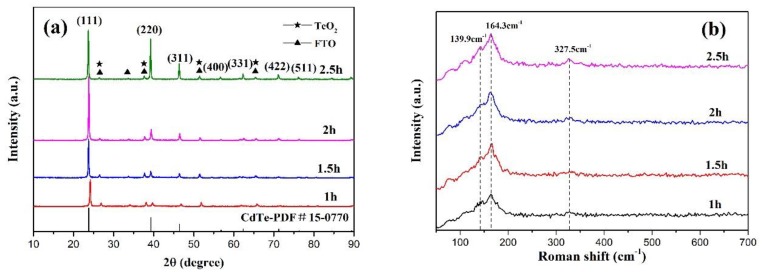
(**a**) XRD patterns of CdTe thin films with deposition time of 1 h, 1.5 h, 2 h, and 2.5 h, respectively; (**b**) Raman spectra of CdTe thin films with deposition time of 1 h, 1.5 h, 2 h, and 2.5 h, respectively.

The structural properties of the CdTe films were further investigated by XRD characterizations, as shown in Figure 2a.

By comparison with the standard card (JCPDS 15-0770), it can be seen that the deposited CdTe thin films have diffraction peaks at 2θ = 23.7°, 39.2°, 46.4°, 56.8°, 62.3°, and 71.2°, which correspond to (111), (220), (311), (400), (331), and (422) planes of CdTe, respectively. Comparing with standard PDF card (JCPDS 42-1445), it is speculated that the peaks at 2θ = 26.8°, 33.6°, 38°, 51.8°, and 65° may be caused by substrate SnO_2_ [19,20]. Peaks at 2θ = 26.8°, 38°, 51.8°, and 65° can also correspond to TeO_2_ (JCPDS 42-1365)_._ The CdTe thin films deposited with 1.5 h have strong diffraction peaks on (111), (220), and (311) planes. Compared with XRD patterns of CdTe thin films deposited with 1 h, the miscellaneous peaks of the diffraction pattern are significantly reduced, which proves that the quality of the prepared CdTe thin films is improved. The CdTe thin films deposited with 2 h have strong diffraction peaks on the (111), (220), and (311) planes, and the diffraction intensity of the miscellaneous peak is further reduced, which proves that the quality of the prepared CdTe thin films is further improved. The XRD analysis of the thin films deposited with 2.5 h shows that the diffraction peaks of planes (111), (220), and (311) are particularly strong, which perfectly corresponds to the peak positions shown in PDF card (JCPDS 15-0770). According to the Debye Scherrer formula [21]:

(1) $$D=\frac{K\lambda }{\beta cos\theta }\;\;\;\;\;\;\;\;\;\;\;\;\;\;\;\;\;\;$$

where *K* is the Scherrer constant, *λ* is the wavelength of X-rays used (*λ* = 0.15 nm), *β* is the full-width at half maximum (FWHM) of the diffraction peaks, and *θ* is Bragg’s angle. The average grain sizes and lattice spacing of (111) plane can be calculated, as shown in Table 1. Compared with the card of PDF (JCPDS 15-0770), it is found that the prepared thin films are of face-centric cubic structure, and the crystalline space group is f-43 m, which is the same as the reported CdTe crystal structure. Raman diffraction patterns of CdTe thin films deposited with different deposition time are shown in Figure 2b. The Raman peaks at the position of 164 cm^−1^ and 327.5 cm^−1^ are consistent with the reported Raman peaks of CdTe thin films [22,23]. All the deposited CdTe thin films also have peaks at 139.9 cm^−1^, which is a combination of TO (CdTe) and elemental Te [24]. It indicates again that Te-rich CdTe thin films have been deposited in our work.

### 3.3. Optical Properties of Deposited CdTe Thin Films

**Figure 3:**
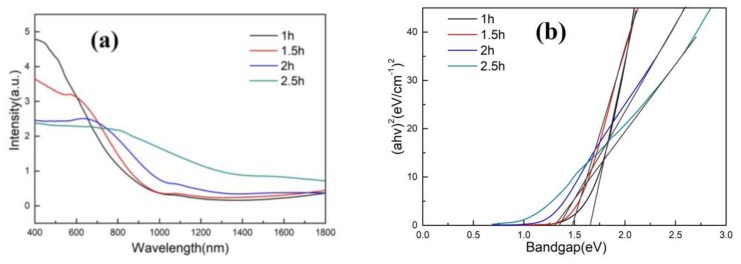
(**a**) UV-Vis diffuse reflection spectra of CdTe thin films with deposition time of 1 h, 1.5 h, 2 h, and 2.5 h, respectively; (**b**) CdTe band gap patterns with deposition time of 1 h, 1.5 h, 2 h, and 2.5 h, respectively.

Figure 3 shows the UV-Vis diffuse-reflection spectra of CdTe thin films with deposition time of 1 h, 1.5 h, 2 h and 2.5 h, respectively. From the images, it can be seen that CdTe thin films have strong absorption of visible light. 

And the optical band gaps of the as-deposited thin films were calculated by diffuse-reflection spectra according to the following equation [25]:

(2) $$\alpha hv=K{\left(hv-E_{g}\right)}^{1/2}$$

In the equation, α was the optical absorption coefficient, *hv* was the photoelectron energy, *Eg* was the band gap width, and *K* was a constant of the material. According to the above equation, CdTe band gaps were calculated as 1.66 eV, 1.48 eV, 1.35 eV, and 1.32 eV, as shown in Figure 3b. The data were consistent with the previous literature reports [12,13]. 

### 3.4. Photoelectrochemical Properties of Deposited CdTe Thin Films

**Figure 4:**
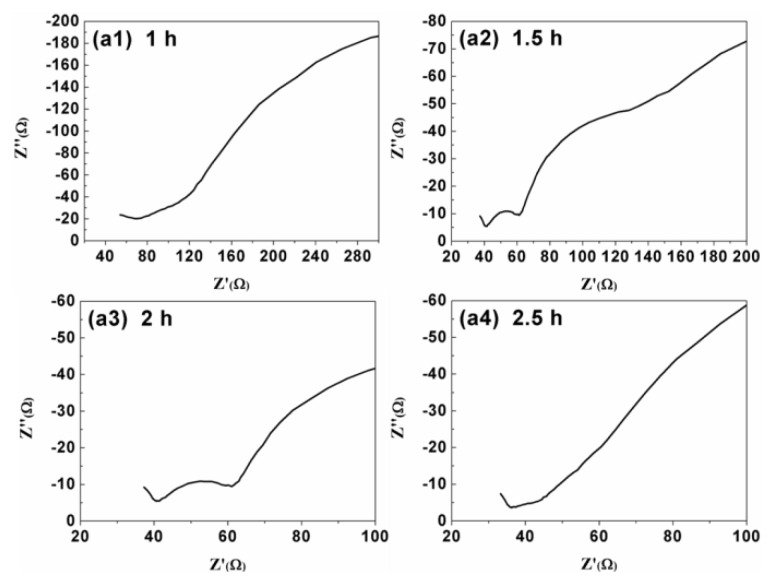
Impedance test images with deposition time of (**a1**) 1 h, (**a2**) 1.5 h, (**a3**) 2 h, and (**a4**) 2.5 h, respectively.

AC impedance test was carried out and the results were shown in Figure 4a1–a4.

The test was performed with a three-electrode configuration. Solution resistance *R_u_* between reference electrode and working electrode, double layer capacitance *C_d_*, and charge transfer resistance *R_ct_* can be obtained from the test. *R_ct_* and *R_u_* of four groups of CdTe thin films in the system were calculated by following formula [26]:

(3) $$Z{}^{\prime }=R_{s}=R_{u}+\frac{R_{ct}}{1+\omega^{2}C_{d}{}^{2}R_{ct}{}^{2}}$$

(4) $$Z{}^{\prime \prime }=\frac{1}{\omega C_{s}}=\frac{\omega C_{d}R_{ct}{}^{2}}{1+\omega^{2}C_{d}{}^{2}R_{ct}{}^{2}}$$

where *ω* was the frequency, *Z’* was the real part of the impedance, Z’’ was the real part of the impedance. As shown in Table 2, the change of *R_u_* is not obvious. Generally speaking, the test solution does not change, nor does *R_u_*. However, there are errors in the measurements, such as the distance between electrodes and the samples in our experiments, the direction of the sample according to the counter electrode, and so on. As deposition time increases, *R_ct_* decreases, which may be caused by film thickening and grain size enlargement.

**Figure 5:**
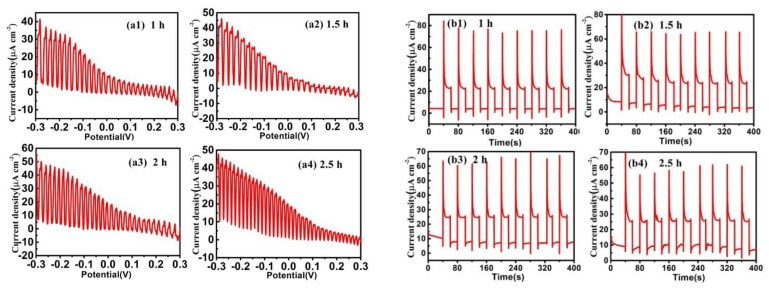
(**a1**–**a4**) Current-potential curves of CdTe thin films with deposition time of 1 h, 1.5 h, 2 h, and 2.5 h, respectively; (**b1**–**b4**) current–time curve of CdTe thin films with deposition time of 1 h, 1.5 h, 2 h, and 2.5 h, respectively.

Under chopped AM 1.5 G light illumination (Newport, Oriel Instruments, optical density = 100 mWcm^−2^), the PEC properties of the films were measured in 0.5 mol/L Na_2_SO_4_ solution by an electrochemical work station (CHI 660B). The tests were measured from −0.3 V to 0.3 V and the scanning rate was 0.01 V/s. Finally, the relationship between current and voltage (I–V curves) was presented in Figure 5a. 

**Figure 6:**
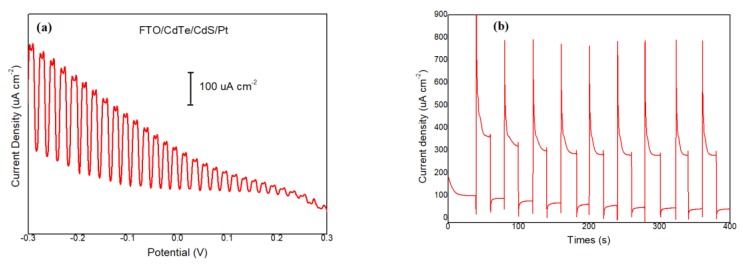
(**a**) Current-potential curves of the Pt/CdS/CdTe/FTO structure; (**b**) current–time curves of the Pt/CdS/CdTe/FTO structure.

It can be seen from the I–V curves that the maximum current difference appears at −0.3 V and this potential is negative, which proves that the prepared CdTe film is p-type material. In Figure 5b, with the increase of deposition time, the photocurrent tends to stabilize at around 25 µAcm^−2^. By comparing the difference of photo and dark current in Table 2, it can be found that the true photo response current first increases and then decreases with the increasing of deposition time. It can be concluded that maximum current can be achieved when the deposition time is 1.5 h and 2 h. The photocurrents are first increased and then decreased, rather than increasing with the deposition time. As known, when the light is irradiated on the semiconductor film, electron and hole pairs are excited inside the semiconductor film. If the film is too thick, the electron–hole pairs will be more likely to recombine before they move to the surface of the films, which will decrease the PEC performance of deposited CdTe thin films [27]. 15 nm CdS film was grown on CdTe film by chemical bath deposition and a 5 nm Pt layer was sputtered on the CdS surface using a sputtering method. The PEC properties of the Pt/CdS/CdTe/FTO structure were characterized, as shown in Figure 6. 

The photocurrents are enhanced greatly and the difference of the light-dark current is increased to 240 µAcm^−2^. The biggest difference of the light–dark current of our deposited CdTe thin film is 20.1 µAcm^−2^. The difference of the light–dark current is enhanced about 12 times after fabrication of the Pt/CdS/CdTe/FTO structure, which demonstrates that this structure can improve the PEC performance of CdTe thin films greatly.

## 4. Conclusions

Te-rich CdTe films have been deposited with the electrodeposition method. SEM studies have shown that the film thickness increases with the deposition time, and the deposition time should be controlled at about 1.5 h–2 h to obtain films with good morphology. The annealed CdTe thin films deposited at 1.5 h–2 h have the largest photocurrent and have good PEC performance. The Pt/CdS/CdTe/FTO structure can improve the PEC properties greatly, and the highest photocurrent of 240 µAcm^−2^ has been achieved. 

## Figures and Tables

**Table 1:** The atomic percents determined by EDS, average grain sizes calculated from XRD data, and the band gaps calculated from UV-Vis diffuse-reflection spectra.

Deposition Time (h)	The Atomic Percent of Cd:Te (%)	Lattice Spacing (nm)	Lattice Parameter (nm)	Average Grain Size (nm)	Band Gap (eV)
1	(46.67 ± 0.51) : (53.33 ± 0.51)	0.3681 ± 0.0002	0.6375 ± 0.0002	30.9 ± 0.1	1.66 ± 0.02
1.5	(47.63 ± 0.51) : (52.37 ± 0.51)	0.3732 ± 0.0002	0.6465 ± 0.0002	36.5 ± 0.1	1.48 ± 0.02
2	(47.85 ± 0.51) : (52.15 ± 0.51)	0.3745 ± 0.0002	0.6486 ± 0.0002	40.1 ± 0.1	1.35 ± 0.02
2.5	(49.33 ± 0.51) : (50.67 ± 0.51)	0.3751 ± 0.0002	0.6497 ± 0.0002	40.1 ± 0.1	1.32 ± 0.02

**Table 2:** Measured AC impedance data and photocurrent data of CdTe prepared at different deposition time.

Deposition Time (h)	*R_u_* (Ω)	*R_ct_* (Ω)	Photocurrent (μAcm^−2^)	Dark Current (μAcm^−2^)	Difference (μAcm^−2^)
1	23.2 ± 0.2	56.3 ± 0.1	22.0 ± 0.1	4.0 ± 0.1	18.0 ± 0.1
1.5	21.3 ± 0.2	36.7 ± 0.1	24.9 ± 0.1	4.8 ± 0.1	20.1 ± 0.1
2	23.8 ± 0.2	17.8 ± 0.1	24.7 ± 0.1	7.4 ± 0.1	17.3 ± 0.1
2.5	20.1 ± 0.2	16.2 ± 0.1	25.2 ± 0.1	8.9 ± 0.1	16.3 ± 0.1
